# HIV leadership programming attendance is associated with PrEP and PEP awareness among young, gay, bisexual, and other men who have sex with men in Vancouver, Canada

**DOI:** 10.1186/s12889-019-6744-y

**Published:** 2019-04-24

**Authors:** Kalysha Closson, Sarah Chown, Heather L. Armstrong, Lu Wang, Nicanor Bacani, Darren Ho, Jody Jollimore, Gbolahan Olarewaju, David M. Moore, Eric A. Roth, Robert S. Hogg, Nathan J. Lachowsky

**Affiliations:** 1 British Columbia Centre for Excellence in HIV/AIDS, 608-1081 Burrard St, Vancouver, BC V6Z 1Y6 Canada; 2 School of Population and Public Health, University of British Columbia, 2206 East Mall, Vancouver, BC V6T 1Z3 Canada; 3 YouthCO HIV & Hep C Society, 205-568 Seymour St., Vancouver, BC V6B 3J5 Canada; 4 Faculty of Medicine, University of British Columbia, 317-2194 Health Sciences Mall, Vancouver, BC V6T 1Z3 Canada; 5 Community-Based Research Centre for Gay Men’s Health, 1007-808 Nelson St., Vancouver, BC V6Z 2H2 Canada; 6 Department of Anthropology, University of Victoria, Cornett Building B228, 3800 Finnerty Road, Victoria, BC V8P 5C2 Canada; 7 Faculty of Health Sciences, Simon Fraser University, 8888 University Drive, Burnaby, BC V5A 1S6 Canada; 8 School of Public Health and Social Policy, University of Victoria, Human and Social Development Building B202, 3800 Finnerty Road, Victoria, BC V8P 5C2 Canada

**Keywords:** Gay, bisexual, and other men who have sex with men, Youth, Leadership, Health promotion

## Abstract

**Background:**

Young gay, bisexual, and other men who have sex with men (YGBM) may have reduced engagement and knowledge of HIV care and biomedical HIV prevention strategies, such as pre-exposure prophylaxis (PrEP), post-exposure prophylaxis (PEP), and Treatment as Prevention (TasP), compared with adult GBM. We sought to understand differences in HIV prevention awareness, health care access, and service utilization between youth (16–29 years) and adult (≥30 year) GBM, as well as factors associated with attendance in HIV leadership programming among YGBM living in the publicly funded PrEP setting of Vancouver, Canada.

**Methods:**

Sexually-active GBM were recruited using respondent-driven sampling (RDS) from February 2012 to February 2015. Participants completed an in-person computer-assisted self-interview every 6 months, up to February 2017, with questions on sociodemographic factors, awareness of biomedical HIV prevention strategies, and an HIV treatment optimism-skepticism scale. Participants were asked if they had ever attended either of two HIV-leadership programs designed for YGBM. Both programs involve multiple GBM-led education and social networking sessions operated by community-based organizations in Vancouver. Multivariable Glimmix confounder models assessed differences between youth and adult GBM. Among younger men, bivariate analyses examined factors associated with HIV-leadership program attendance.

**Results:**

Of 698 GBM who enrolled in the longitudinal study, 36.8% were less than 30 years old at the first study visit. After controlling for gender identification, sexual orientation, HIV status, and income in the past 6 months, younger GBM (*n* = 257/698) had lower awareness of biomedical HIV prevention strategies and less HIV treatment optimism compared with older GBM (*n* = 441/698). Among younger GBM who attended HIV-leadership programs (*n* = 50), greater awareness of biomedical HIV prevention strategies and higher HIV treatment optimism were reported, compared with non-attendees.

**Conclusion:**

Younger GBM, who are disproportionately affected by the HIV epidemic, are less aware of new prevention technologies than older GBM, but attending peer-based HIV-leadership programs ameliorates age-disparities in HIV-prevention knowledge and treatment optimism.

## Background

Young adulthood is a life stage characterized by self and sexual exploration which can lead to higher incidence of sexually transmitted infections (STIs), including human immunodeficiency virus (HIV), higher substance use, and greater mental health concerns. Today, young gay, bisexual, and other men who have sex with men (YGBM) are initiating sexual activity in an era with increased access to HIV antiretroviral therapy used now for both treatment and prevention. However, YGBM continue to represent a key population in the global HIV epidemic [1]. In Canada, GBM represented 44.1% of all new infections in 2016, with over half of all new infections among those aged 15–29 years old attributed to GBM [2]. Moreover, evidence suggests that HIV incidence may be increasing among younger generations of men [3].

Prior to relatively recent improvements in antiretroviral-based HIV prevention strategies, condom promotion and seroadaptive behavioral strategies were the main HIV prevention mechanisms promoted to and used by GBM [4]. These strategies continue to play a key part in comprehensive HIV prevention strategies, but new HIV transmissions persist [4, 5]. Prevention frameworks, including Coates’ 2008 highly active HIV prevention framework [6], acknowledge the need for multiple strategies including: 1) behavioral adaptations such as serosorting, seropositioning, and viral load sorting, 2) antiretroviral treatment for those living with HIV and campaigns like “undetectable equals untransmittable” (U=U) that highlight people with undetectable HIV viral loads cannot transmit the virus, 3) antiretroviral prevention interventions like pre-exposure prophylaxsis (PrEP) and post-exposure prophylaxsis (PEP), and 4) social justice and human rights, such as those that provide protection for all sexual orientations, gender identities, and gender expressions. Collectively, these strategies would reduce HIV transmission among GBM if the options are implemented equitably [1, 6, 7]. Unfortunately, important strides in prevention options are co-occurring against a background of persistent HIV stigma and homophobia [8–10]. Thus, despite increasing visibility of some gay men’s lives and greater awareness of the inequities experienced by GBM, frameworks for highly active HIV prevention are rarely adopted within health promotion initiatives. These realities have important implications for GBM in general, and particularly for younger GBM who are initiating their sexual lives in a climate of shifting societal norms and evolving HIV prevention technologies.

In British Columbia, Canada PrEP is currently available at no cost to eligible individuals based on clinical guidelines, one criterion being a score of 10 or greater on the HIV incidence risk index for men who have sex with men (HIRI-MSM); PrEP eligibility favours younger men, as GBM aged between 18 and 28 years receive eight points on the HIRI-MSM tool just due to age [11]. Despite increased diversity in potential HIV prevention and treatment strategies, YGBM may not be receiving adequate comprehensive sexual education and community support to effectively use them. As such, youth advocacy groups have stated that they want sexual education that is standardized, fun, relevant, and delivered by allies who are comfortable talking to young people about sex [12]. Across a number of provinces in Canada, limited information is provided to students regarding diversities in sexual orientation and gender identity [13]. In the classroom, education on sexual practices predominantly discusses penile-vaginal sex, with few opportunities for students to learn and ask questions about other sexual behavior [14]. Poor access to comprehensive sex education that addresses the unique sexual health needs of YGBM, may result in reduced awareness of important components of sexual health, including testing, biomedical prevention strategies including PrEP, and treatment options for STIs and HIV [15, 16]. Without adequate awareness of the combination prevention options, YGBM will likely continue to be at increased risk of acquiring HIV. In BC, there has been increasing efforts to address school safety with supportive policies; these have found to be effective at improving both the health outcomes of sexual and gender minority youth students, as well as other students [17].

Historically, evidence-based interventions for sexual health among GBM have focused on individual-level behavior change and one example is Mpowerment [18, 19]. Mpowerment is an evidence-based behavioral intervention developed at the University of California-San Francisco in the early 2000s [20]. Since 2012, this program has been implemented in Vancouver by YouthCO, a youth-led community-based organization for youth living with and affected by HIV and Hepatitis C (www.youthco.org/mpowerment). At YouthCO, all programming takes place within the values of anti-oppression. The Mpowerment Project leverages social networks to provide accurate, stigma-free information about sexual health to sexually active YGBM. Led by YouthCO staff and a dedicated group of volunteers known as Core Group, MpowermentYVR is continuously adapted to respond to local needs and integrates a highly-active HIV prevention strategy into the programming through sharing information, access to HIV treatment and prevention tools (e.g., condoms, testing), and skills building to help HIV-positive and HIV-negative participants navigate their sexual lives and the myriad of social factors that affect them (e.g, substance use, mental health, and social connection). This program offers alternative social spaces and opportunities to cultivate friendships and community outside of the typically sexualized “gay bar” setting.

Another youth leadership program for YGBM in Vancouver is Totally Outright [21], which is funded by the Public Health Agency of Canada. The program was launched in 2005 by the Community-Based Research Centre, a Vancouver-based non-profit organization working to strengthen health outcomes for GBM [21], and consists of a 40-h leadership course including a series of workshops, a fieldwork assignment, a group project, and social activities which together aim to develop a corps of sex-savvy YGBM who would educate others about highly-active HIV prevention, sexual and mental health as well as emerging concepts like harm reduction, seroadaptive strategies, social justice, and anti-oppression. Applying both peer-based and intergenerational learning has proven to be an effective model and the program continues to run annually in Vancouver, offered by Health Initiative for Men (HIM), and across Canada by other front-line organizations in a variety of cities including Edmonton, Calgary, Winnipeg, Toronto, and Halifax. Both MpowermentYVR and Totally Outright aim to improve the sexual health literacy, knowledge, and awareness of HIV prevention and treatment among YGBM who are disproportionately affected by the epidemic.

Given the disproportionate risk of HIV among YGBM, we sought to understand gaps in HIV prevention uptake by examining differences in HIV prevention awareness, health care access, and service utilization between youth and adult GBM. Additionally, we sought to explore factors associated with participating in these two youth-focused GBM-led HIV leadership programs among YGBM living in Vancouver, Canada. We hypothesized that younger GBM would have less awareness about HIV prevention technologies than older GBM. We further hypothesized that YGBM who participated in an HIV leadership program would have increased awareness of biomedical HIV prevention technologies and more HIV treatment optimism, relative to non-attendees.

## Methods

### Study sample

We use an observational study design to address our study aims in order to evaluate the real-world uptake, effectiveness and impact of these programs. Sexually active GBM were recruited into a longitudinal cohort study using respondent-driven sampling (RDS) from February 2012 to February 2015, with follow-up visits occurring every 6 months until February 2017. Details of the study methodology have been previously reported [22]. In brief, GBM were eligible for participation if they identified as a man, including trans men, reported having sex with another man in the past 6 months, were at least 16 years of age, lived within Metro Vancouver, and were able to complete a questionnaire in English. The study office was located in Vancouver’s West End, Vancouver’s traditional gay neighborhood. At each study visit, participants completed a computer-assisted, self-administered questionnaire on demographics, attitudes, sexual behavior, HIV prevention practices, and community leadership program utilization. The Momentum study employed a nurse that was specifically trained in public health and STI certified practice [23]. At each visit, the nurse administered a brief questionnaire, the HIV and STI testing, and provided referrals to primary care, community services or other resources for any issues that arose.

Participants received a $50 honorarium for completing each study visit (or the equivalent value in draw tickets for a larger prize) and an additional $10 for each subsequent eligible participant they recruited (to a maximum of six). Written informed consent was obtained from all participants prior to participation. All study activities were approved by the research ethics boards of the University of British Columbia, the University of Victoria, and Simon Fraser University.

### Dependent variable

We examined select differences between young GBM (aged 16–29) and older GBM (≥30 years). We chose to compare those under 30 to those 30 and over (time-dependent) to be consistent with past research conducted among YGBM, and to match the Commonwealth definition of youth [24, 25].

We further conducted a sub-analysis that included all study participants < 35 years who were asked about participation in two HIV leadership programs (MpowermentYVR and Totally Outright). The wider age range was used to accommodate eligibility in the community programs across the two-year data collection period (2015–2017) and to better capture the duration these programs have been running in Metro Vancouver (e.g. Mpowerment was launched in 2012 5 years prior to the end of data collection). Questions regarding HIV leadership attendance were added later in the study and thus were collected over a two-year study period from 2015 to 2017. Program attendance was measured as a time varying visit-level outcome where participants could report no attendance in one visit but report attendance in a following visit.

### Independent variables

In order to assess differences between younger and older GBM, we examined the association between a number of demographic, sexual behavior, prevention strategies, awareness of biomedical HIV prevention options, and health seeking behaviors with age group (youth vs. adult GBM).

Demographic variables were controlled for in the analyses. These included: gender identity (gender minority, cis male), sexual orientation (gay, bisexual, other), race/ethnicity (White, Indigenous, Asian, other), lab-confirmed HIV serostatus (negative, positive), formal education (did not complete high school, completed high school), and annual income (<$30,000 CAD, $30,000 CAD-$59,999 CAD, ≥$60,000 CAD).

Sexual behavior items included having a current regular partner, number of sexual partners in the past 6 months, and engaging in any escort/sex work in the past 6 months. HIV risk behavior was assessed using a 4-level categorical variable: only condom-protected anal intercourse in the past 6 months (referent) compared with not engaging in anal sex, any condomless anal sex with an opposite or unknown HIV status partner, or condomless anal sex only with same HIV serostatus partners. Participants were asked about five different HIV prevention practices in the past 6 months: consistent condom use, seropositioning (i.e., selecting anal sex position based on HIV status); serosorting (i.e., selective condom non-use with sexual partners with the same HIV status); viral load sorting (i.e., selective condom non-use when the HIV-positive partner is on treatment or has an undetectable viral load); and asking partners their HIV status.

Awareness of HIV prevention strategies was determined by asking participants two items to assess whether or not they had heard of PrEP and PEP (No vs. Yes). Participants’ attitudes towards HAART were assessed using Van de Ven’s HIV treatment optimism-skepticism scale [26]. Participants responded using a 4-point Likert scale to indicate agreement with 12 statements regarding viral load testing and HIV treatment (e.g., “New HIV treatments will take the worry out of sex”), with higher scores indicating greater treatment optimism (range = 12–48; study Cronbach alpha = 0.85).

Items related to health care and service utilization asked participants about whether they had been tested for any STIs as well as if they had been diagnosed with any STI in the past 6 months (P6M). Participants were also asked about whether they have a general practitioner (GP) and for those that did, if they had told their GP about their sexual orientation.

### Statistical analysis

Descriptive statistics are presented by age category. Bivariate analyses were performed using Pearson’s chi-squared or Fisher’s exact test for categorical variables and Wilcoxon rank sum test for continuous variables to examine differences between GBM under 30 compared with those 30 and older.

Unadjusted and adjusted generalized linear mixed models for longitudinal data were used to account for RDS chain and participant (from all visits) clustering in order to assess differences in awareness, access, and service utilization (i.e., having a GP) between younger (18–29 years old) and older GBM (≥30). Adjusted models controlled for HIV status, income, gender, and sexual orientation.

Among participants under the age of 35 years who were asked about participating in two HIV leadership programs, unadjusted generalized linear mixed models were used to examine factors associated with ever attended (at any visit) a YGBM HIV prevention leadership program. Statistical interactions were tested to examine whether HIV-status modified the effect of each independent variable on program participation at the univariable level.

## Results

Of 698 GBM who enrolled in the longitudinal study, 36.8% were less than 30 years old at first visit. Table 1 presents baseline sociodemographic, sexual health attitudes and practices, and sexual behavior characteristics by age group. It should be noted that even though YGBM aged 16–17 were eligible for participation, the youngest participants were 18 years old. The majority of participants were White (75.5%), with no statistically significant difference in ethnicity breakdown by age group. YGBM were more likely than older GBM to identify as a gender minority (5.1% versus 0.7%; *p* < 0.001), to identify as a sexual minority other than gay or bisexual (10.5% versus 3.9%, *p* = 0.001), to be HIV-negative (95.7% versus 56.9%; *p* < 0.001), to have an annual income <$30,000 CAD (72.0% versus 54.9%, < 0.001), and to be single (65.8% versus 57.8%). In the 6 months prior to the baseline study visit, YGBM were more likely to report using consistent condom use (67.6% versus 45.6%) and asking their partner about their HIV status (65.2% versus 55.8%, *p* = 0.015) as HIV prevention strategies compared with older GBM. YGBM were less likely to report any condomless anal sex with serodiscordant or unknown status partners (32.0% vs. 44.4%, *p* = 0.002), and less likely to use seropositioning (20.7% versus 34.4%; *p* < 0.001) and viral load sorting (7.4% versus 25.7%, *p* < 0.001) as HIV prevention strategies. YGBM were less likely to be aware of PrEP (14.4% versus 29.4%, *p* < 0.001) and they had significantly lower HIV treatment optimism (mean score: 24 versus 26; *p* < 0.001).

**Table 1:** Baseline sociodemographic, sexual health attitudes and practices, and sexual behavior characteristics between youth (16–29) and adult (≥30) GBM in Vancouver, BC (*n* = 698)

	Younger (*n* = 257) N (%)	Older (*n* = 441) N (%)	*P*-value
Demographics			
Gender Identity			< 0.001
Male	244 (94.9)	438 (99.3)	
Transman/Intersex	13 (5.1)	3 (0.7)	
Sexual Orientation			0.001
Gay	216 (84.1)	384 (87.1)	
Bisexual	14 (5.5)	40 (9.1)	
Other	27 (10.5)	17 (3.9)	
Ethnicity			0.098
White	197 (76.7)	330 (74.8)	
Indigenous	30 (11.7)	39 (8.8)	
Asian	8 (3.1)	32 (7.3)	
Other	22 (8.6)	40 (9.1)	
HIV Status			< 0.001
Negative	246 (95.7)	251 (56.9)	
Positive	11 (4.3)	190 (43.1)	
Formal Education			0.592
Did not complete high school	17 (6.6)	34 (7.7)	
Completed high school	240 (93.4)	407 (92.3)	
Annual Income			< 0.001
< $30,000	185 (72.0)	242 (54.9)	
$30,000–$59,999	61 (23.7)	126 (28.6)	
$60,000 or more	11 (4.3)	73 (16.6)	
Relationship Status			0.038
Single	169 (65.8)	255 (57.8)	
Partnered	88 (34.2)	186 (42.2)	
Behavior			
Number of Male Sex Partners, P6M: median, Q1-Q3	5 (3–11)	5 (2–19)	0.340
Sexual Risk, P6M			0.002
No anal sex	27 (10.7)	55 (12.8)	
Only condom-protected anal intercourse	69 (27.3)	78 (18.1)	
Any condomless anal sex, but only seroconcordant	76 (30.0)	106 (24.7)	
Any serodiscordant condomless anal sex	81 (32.0)	191 (44.4)	
Any Escort/Sex Work			< 0.001
No	223 (86.8)	350 (79.4)	
Yes, in P6M	20 (7.8)	24 (5.4)	
Yes, but not in P6M	14 (5.5)	67 (15.2)	
Prevention Practice, P6M: Consistent Condom Use			< 0.001
No	83 (32.4)	239 (54.4)	
Yes	173 (67.6)	200 (45.6)	
Prevention Practice, P6M: Seropositioning			< 0.001
No	203 (79.3)	288 (65.6)	
Yes	53 (20.7)	151 (34.4)	
Prevention Practice, P6M: Serosorting			0.384
No	155 (60.5)	251 (57.2)	
Yes	101 (39.5)	188 (42.8)	
Prevention Practice, P6M: Viral Load Sorting			< 0.001
No	237 (92.6)	326 (74.3)	
Yes	19 (7.4)	113 (25.7)	
Prevention Practice, P6M: Ask Partner Their HIV Status			0.015
No	89 (34.8)	194 (44.2)	
Yes	167 (65.2)	245 (55.8)	
Awareness and Attitudes			
Heard of TasP			0.009
No	139 (54.1)	193 (43.9)	
Yes	118 (45.9)	247 (56.1)	
Heard of PrEP			< 0.001
No	178 (85.6)	243 (70.6)	
Yes	30 (14.4)	101 (29.4)	
Heard of nPEP			0.078
No	100 (48.1)	139 (40.4)	
Yes	108 (51.9)	205 (59.6)	
HIV Treatment Optimism Scale Score			< 0.001
median, Q1-Q3	24 (21–27)	26 (21–30)	
Health Care			
Any STI Testing, P6M			0.205
No	98 (43.0)	149 (37.8)	
Yes	130 (57.0)	245 (62.2)	
Any STI Diagnosis, P6M			0.774
No	221 (88.0)	364 (88.8)	
Yes	30 (12.0)	46 (11.2)	
Have a General Practitioner (GP)			< 0.001
No	138 (54.3)	82 (18.8)	
Yes	116 (45.7)	355 (81.2)	
Told GP about sexual orientation “out”^a^			< 0.001
No	38 (32.8)	31 (8.7)	
Yes	78 (67.2)	324 (91.3)	

*P6M* past 6 months, *TasP* treatment as prevention, *PreP* Pre-exposure Prophylatic, *nPEP* Post-exposure prophylactic, *STI* Sexually Transmitted Infection, *HIV* Human Immunodeficiency Virus, *GP* General Practioner

^a^Only asked to those that reported having a GP

Table 2 presents unadjusted and adjusted generalized linear mixed models for comparisons between younger and older GBM. After controlling for gender identification, sexual orientation, HIV status, and income, consistent condom use as an HIV prevention strategy (aOR = 1.45, 95%CI = 1.07–1.98), and reporting sex/escort work in the past 6 months (aOR = 2.64, 95%CI = 1.32–5.27) were positively associated with younger age. Having a GP (aOR = 0.25, 95%CI = 0.15–0.41) was negatively associated with younger age. Among GBM who had a GP, participants who have not disclosed their sexual orientation (aOR = 0.35, 95%CI = 0.17–0.72) were more likely to be younger. Participants who have heard of PrEP (aOR = 0.43, 95%CI = 0.31–0.59) and PEP (aOR = 0.53, 95%CI = 0.36–0.77), and had higher HIV treatment optimism scores (aOR = 0.93, 95%CI = 0.90–0.96; per 1 point scale increase), were less likely to be young.

**Table 2:** Unadjusted and adjusted odd ratios for differences between youth and adult GBM (*n* = 698)

	Unadjusted Odds Ratio (OR) (95%CI)	Adjusted Odds Ratio (aOR) (95%CI)
Demographics		
Gender Identity		
Male	1.00	1.00
Trans-man/Intersex	**5.12 (1.93–13.62)**	**3.33 (1.14–9.74)**
Ethnicity		
White	1.00	1.00
Asian	0.74 (0.30–1.85)	0.75 (0.31–1.83)
Indigenous	0.26 (0.06–1.10)	**0.19 (0.04–0.84)**
Other	0.83 (0.31–2.23)	0.67 (0.24–1.87)
Sexual Orientation		
Gay	1.00	1.00
Bisexual	0.86 (0.40–1.83)	0.71 (0.31–1.60)
Other	**2.24 (1.24–4.04)**	1.52 (0.83–2.80)
HIV Status		
Negative	1.00	1.00
Positive	**0.03 (0.01–0.06)**	**0.02 (0.01–0.04)**
Annual Income		
< $30,000	1.00	1.00
$30,000–$59,999	**0.45 (0.31–0.66)**	**0.34 (0.23–0.50)**
$60,000 or more	**0.10 (0.05–0.20)**	**0.06 (0.03–0.12)**
Behavior		
Recent Male Sex Partners, P6M: per 10 units increase	0.98 (0.95–1.02)	1.00 (0.97–1.04)
Sexual Risk, P6M		
No anal sex	**0.65 (0.43–0.99)**	**0.61 (0.39–0.97)**
Only condom-protected anal intercourse	1.00	1.00
Any condomless anal sex, but only seroconcordant	1.02 (0.72–1.44)	1.25 (0.83–1.87)
Any serodiscordant condomless anal sex	**0.59 (0.42–0.83)**	0.73 (0.50–1.08)
Injection drug use, P6M		
No	1.00	1.00
Yes	**0.53 (0.31–0.92)**	0.79 (0.41–1.53)
Any Escort/Sex Work		
No	1.00	1.00
Yes, in P6M	**2.31 (1.28–4.18)**	**2.64 (1.32–5.27)**
Yes, but not in the P6M	1.55 (0.67–3.56)	1.80 (0.70–4.66)
Prevention Practice, P6M: Consistent Condom Use		
No	1.00	1.00
Yes	**1.82 (1.35–2.46)**	**1.45 (1.07–1.98)**
Prevention Practice, P6M: Sero- positioning		
No	1.00	1.00
Yes	**0.53 (0.37–0.76)**	**0.55 (0.37–0.81)**
Prevention Practice, P6M: Viral Load Sorting		
No	1.00	1.00
Yes	**0.44 (0.27–0.71)**	0.75 (0.44–1.26)
Ever heard of PrEP		
No	1.00	1.00
Yes	**0.35 (0.27–0.46)**	**0.43 (0.31–0.59)**
Ever heard of nPEP		
No	1.00	1.00
Yes	**0.44 (0.32–0.62)**	**0.53 (0.36–0.77)**
HIV Treatment Optimism Scale Score per one score increase	**0.89 (0.86–0.91)**	**0.93 (0.90–0.96)**
Health Care		
Have a GP		
No	1.00	1.00
Yes	**0.13 (0.08–0.22)**	**0.25 (0.15–0.41)**
“Out” to GP^a^		
No	1.00	1.00
Yes	**0.14 (0.07–0.28)**	**0.35 (0.17–0.72)**

Models adjusted for gender, sexual orientation, HIV status, and income

*P6M* past 6 months, *TasP* treatment as prevention, *PreP* Pre-exposure Prophylactic, *nPEP* Post-exposure prophylactic, *STI* Sexually Transmitted Infection, *HIV* Human Immunodeficiency Virus, *GP* General Practitioner

^a^Only asked to those that reported having a GP

Bold are statistically significant *p* <0.05

**Fig. 1:**
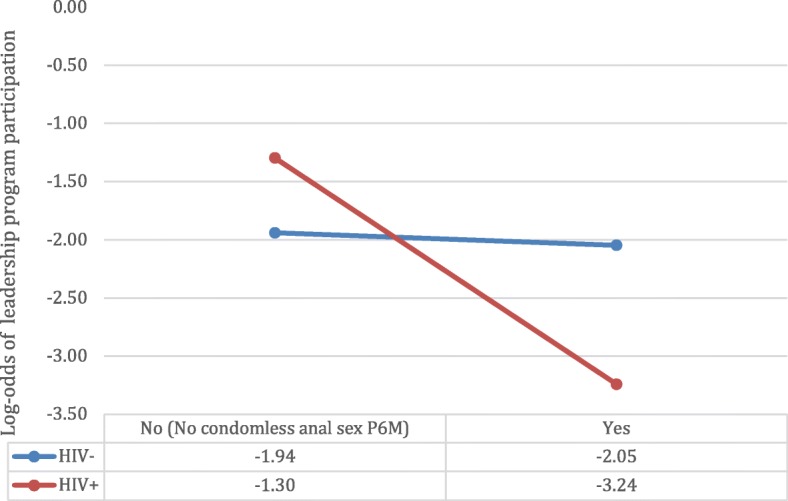
Interaction between HIV status and any recent condomless anal sex with a serodiscordant or unknown status partner (yes) versus none on the outcome of HIV leadership program attendance

Table 3 presents a sub-analysis of participants aged < 35 years at baseline (*n* = 246, 35.2% of the entire sample) who were indicated at 889 study visits about whether or not they had ever or recently participated in either of the two Vancouver-based leadership programs. Fifty participants (20.3% of age sub-sample) reported ever or recently participating. Percentages reported in Table 3 show a visit-level comparison between visits where YGBM indicated program participation and all visits where no participation was recorded. Those that reported ever participating in a leadership program were significantly more likely to have used serosorting as an HIV prevention strategy in the P6M (OR = 1.55, 95%CI = 1.03–2.35), to have heard of PrEP (OR = 2.19, 95%CI = 1.12–4.29), to have heard of PEP (OR = 2.89, 95%CI = 1.20–6.92), and to have higher HIV treatment optimism scores (OR = 1.06 per 1 score increase, 95%CI = 1.01–1.12). The interaction between HIV status and any condomless anal sex with an opposite/unknown status partner in the P6M (CAS) was significant with respect to leadership program attendance; there was a significant association between CAS and program participation for HIV-positive GBM, and no significant difference found for HIV-negative men. HIV-positive participants who reported recent CAS were more likely to have attended an HIV leadership program compared with HIV-positive GBM who did not attend leadership programs (aOR = 0.14, 95%CI = 0.03–0.61); there was no significant difference for HIV-negative participants in CAS between leadership program attendance or not (aOR = 0.90, 95%CI = 0.43–1.87; aOR ratio = 0.16, 95%CI = 0.03–0.80) (Fig. 1).

**Table 3:** Socio-demographic, sexual health attitudes and practices, and sexual behavior characteristics between youth (aged 18 to 30 at baseline participating and not participating in youth-oriented HIV leadership programs in Vancouver, BC (*n* = 246; 889 visits)

Characteristics	Use of youth leadership programming (at each visit)?		Unadjusted Odds Ratio (OR) and 95% Confidence Interval (CI)
	No (*n* = 717)	Yes (*n* = 172)	
	N (%)	(%)	
Demographics			
Gender Identity			
Male	672 (93.7)	168 (97.7)	1.00
Trans-man/Intersex	45 (6.3)	4 (2.3)	0.38 (0.03–5.37)
Ethnicity			
White	515 (71.8)	130 (75.6)	1.00
Asian	97 (13.5)	18 (10.5)	0.74 (0.24–2.29)
Indigenous	36 (5.0)	14 (8.1)	1.54 (0.39–6.05)
Other	69 (9.6)	10 5.8	0.57 (0.13–2.47)
Sexual orientation			
Gay	598 (83.4)	141 (82.0)	1.00
Bisexual	42 (5.9)	4 (2.3)	0.51 (0.10–2.49)
Other	77 (10.7)	27 (15.7)	1.74 (0.57–5.37)
HIV status			
HIV Negative	640 (89.3)	162 (94.2)	1.00
HIV Positive	77 (10.7)	10 (5.8)	0.41 (0.06–2.63)
Annual income			
Less than $30,000	350 (49.0)	98 (57.0)	1.00
$30,000 to $59,999	247 (34.6)	57 (33.1)	1.14 (0.57–2.28)
$60,000 and more	118 (16.5)	17 (9.9)	0.57 (0.17–1.87)
Behavior			
Sexual Risk, P6M			
No anal sex	117 (16.5)	26 (15.1)	0.60 (0.30–1.22)
Only condom-protected anal intercourse	142 (20.1)	30 (17.4)	1.00
Any condomless anal sex, but only seroconcordant	216 (30.5)	75 (43.6)	1.25 (0.75–2.09)
Any serodiscordant condomless anal sex	233 (32.9)	41 (23.8)	0.69 (0.35–1.34)
Any escort/sex work			
No	695 (96.9)	158 (91.9)	1.00
Yes P6M	22 (3.1)	14 (8.1)	2.62 (0.80–8.61)
Yes but not P6M	0 (0.00)	0 (0.00)	-
Prevention Practice P6M: Consistent condom use			
No	278 (39.0)	70 (40.9)	1.00
Yes	435 (61.0)	101 (59.1)	0.89 (0.59–1.34)
Prevention Practice P6M: Seropositioning			
No	512 (71.8)	128 (74.9)	1.00
Yes	201 (28.2)	43 (25.1)	0.95 (0.54–1.67)
Prevention Practice P6M: Serosorting			
No	406 (56.9)	72 (42.1)	1.00
Yes	307 (43.1)	99 (57.9)	**1.55 (1.03–2.35)**
Prevention Practice P6M: Viral load sorting			
No	587 (82.3)	148 (86.6)	1.00
Yes	126 (17.7)	23 (13.5)	0.68 (0.32–1.44)
Prevention Practice P6M: Ask partner their HIV status			
No	243 (34.1)	70 (40.9)	1.00
Yes	470 (65.9)	101 (59.1)	0.79 (0.52–1.21)
Attitudes and awareness			
Ever heard of PrEP			
No	131 (18.3)	18 (10.5)	1.00
Yes	586 (81.7)	154 (89.5)	**2.19 (1.12–4.29)**
Ever heard of nPEP			
No	96 (13.4)	7 (4.1)	1.00
Yes	621 (86.6)	165 (95.9)	**2.89 (1.20–6.92)**
HIV Treatment Optimism Scale Score per one score increase	27 (24–31)	28 (25–30)	**1.06 (1.01–1.12)**
Health Care			
Have a GP			
No	271 (38.3)	81 (48.2)	1.00
Yes	437 (61.7)	87 (51.8)	0.71 (0.33–1.53)
Out to GP			
No	87 (19.9)	29 (33.3)	1.00
Yes	350 (80.1)	58 (66.7)	0.51 (0.13–2.06)
Ever HPV vaccination			
No	693 (98.4)	162 (96.4)	1.00
Yes	11 (1.6)	6 (3.6)	1.48 (0.57–3.83)

Bold are significant at *p*<0.05

## Discussion

GBM in our study who reported consistent condom use and were less likely to use HIV seroadaptive preventive practices such as serosorting and viral load sorting were more likely to be young. Participants less aware of biomedical HIV prevention strategies such as PrEP and had less HIV treatment optimism were also more likely to be younger. GBM without a GP, and among those who had a GP but were not ‘out’ to their GPs were also more likely to be young. Approximately 1 in five YGBM had participated in youth-led HIV prevention leadership programming such as MpowermentYVR or Totally Outright in Vancouver. YGBM who participated in these programs were significantly more likely to be aware of PrEP and PEP, and had higher HIV treatment optimism. Moreover, only among YGBM living with HIV did we find that HIV leadership programs attendance was associated with lower odds of condomless anal sex with serodiscordant or unknown HIV status partner. YGBM living with HIV face unique challenges navigating their sexual lives compared with HIV-negative youth peers, and may be choosing to employ serosorting or avoid anal sex altogether.

Despite previous studies in the US highlighting increased condomless sex among YGBM [27], encouragingly we found that 68% of YGBM in Momentum reported using consistent condom use as an HIV prevention strategy, which was significantly higher than older GBM in this study (45.6%). Condom use promotion continues to be a key message in sexual education for young people, including YGBM, which may help explain why condom use seems to have been embraced by our younger participants [28].

Reduced PrEP and PEP awareness among YGBM in our study is an important finding given that previous work highlighted that YGBM in Vancouver who were less aware of PrEP were HIV-negative, had poorer access to condoms, and preferred receptive anal sex, indicating multiple factors for increased HIV acquisition [29]. Attending HIV leadership programming ameliorated YGBM’s awareness disparities with respect to PrEP and PEP. In our sub-analysis, YGBM who attended youth-led HIV leadership and prevention programs were significantly more likely to be aware of PrEP and PEP than those who did not attend. Previous studies have examined the impact of Mpowerment, highlighting that program attendance has been associated with increased condom use [20]. To our knowledge this was the first study to look at HIV leadership programming attendance and PrEP or PEP awareness. Our findings highlight that spaces and programming that are run by, with, and for youth may be more accessible and beneficial for YGBM. There is a need for these programs to be sustained and expanded in order to reach more YGBM. In addition to PrEP, YGBM living with HIV would especially benefit from additional education and support around newer biomedical advancements like U=U [6].

Young adulthood is often a life stage in which individuals are at their healthiest [30, 31]. Moreover, there are few resources for youth specific health care delivery [32, 33]. In the current study, we found that only half of YGBM had a GP, which is lower than the national average of youth accessing primary care in Canada [34] and significantly lower than older GBM in our study. This indicates a structural barrier for YGBM to access biomedical treatment and prevention options from a physician. Previous Canadian research has highlighted that young adults (18–30) have a higher proportion of unmet health needs largely due to the inability to attain a primary care provider who is accepting new patients [35]. Younger individuals are less likely to seek primary care in general, and in Canada there is a large shortage of doctors accepting new patients. Moreover, many YGBM in Vancouver may have recently moved to the city from other parts of Canada or immigrated from other countries [36].

Beyond the systematic lack of access to GPs, we found that YGBM with GPs were less likely to be out to their GPs. Previous research among GBM in Vancouver has found that GBM who did not disclose their sexual orientation and/or practices were less likely to have been tested for HIV [37]. YGBM may choose to not disclose their sexual health behaviours to their physicians for numerous reasons. YGBM who may be less likely to access primary health care and be out to health providers due to stigma and discrimination and may not feel comfortable discussing sexual health concerns with their primary health care provider [38]. Moreover, primary care providers themselves may not feel comfortable discussing sexual health concerns with their patients [39]. Experiencing heteronormative assumptions within healthcare settings where GBM seek sexual health services profoundly influences young men’s experiences of these services [38].

These findings have important implications for health care and education systems. Positively, findings surrounding consistent condom use indicate that many YGBM are taking precaution to reduce passing HIV and other STIs to their partners. However, critical disparities in HIV prevention awareness and access to primary health care that is culturally competent in terms of sexuality remain. Health service delivery should consider implementing youth- and LGBTQ-friendly sexual health spaces in which appropriate sexual healthcare and education can be delivered to YGBM in an effort to reduce HIV transmission at younger ages. Moreover, low PrEP awareness and HIV treatment optimism among YGBM should be addressed through increased relevant and comprehensive sexual education in schools that is grounded in the lived realities of YGBM [40]. Youth, regardless of sexual orientation, can benefit from increased knowledge and awareness of the different HIV prevention tools and resources that are available to them and their peers [1]. Moreover, to ensure sexual health literacy begins early, youth-friendly and inclusive spaces for sexual health should be included in the classroom. In order to improve inclusivity, there is a need to reform health care service delivery and sexual health education that is often heteronormative [38]. One way in which to facilitate improved acceptability and access for YGBM would be to cultivate professional allies in health care and educational settings [41]. While these systems changes are being implemented, investing in youth-led programming outside of formal health care and educational settings is essential to promote sexual health for YGBM.

### Strengths and limitations

The use of RDS-sampling within our study allowed for a more diverse, if not representative, cohort of GBM in a setting where HIV treatment is available at no cost [42, 43]. While our sub-analysis of YGBM attending leadership programs had limited statistical power, it leveraged a population-based cohort to provide external empirical evidence as to some of the impacts of these programs. Due to small counts in many of the ethnicity groups within this study we were unable to meaningfully discuss the realities of YGBM of colour. We acknowledge that there is a need for increased research to focus on the needs and strengths of GBM who are Indigenous and/or of colour internationally and in Canada. The observational design provides a real-world perspective of effectiveness, although cannot produce the specific efficacy data that randomized trials would yield. Given the positive impact on PrEP and PEP awareness among those YGBM who attended leadership programming, future studies with different designs and sampling should further examine the impact of participation in these programs.

## Conclusion

Our study highlights that participants that had less awareness of new prevention technologies tended to be younger than those who had higher awareness. However, participation in GBM-led HIV leadership programs was associated with increased HIV-prevention knowledge and treatment optimism. Findings suggest that given limited access to primary care alongside potentially inadequate comprehensive sexual health education, community-led and youth-friendly sexual health programming are beneficial for improving YGBM’s awareness of different HIV prevention mechanisms. Efforts need to continue to work  with YGBM to address gaps in sexual health education in schools, and to explore ways in which school- and community-level programming can increase sexual health literacy as well as access to and uptake of necessary HIV prevention resources.
